# No evidence of major integrative brain clearance impairment in an Alzheimer´s disease model of transgenic APPswe/PS1dE9 mice

**DOI:** 10.1186/s12987-026-00872-9

**Published:** 2026-09-05

**Authors:** Luisa Müller, Charlotte Köhler, Tobias Lindner, Stefan Polei, Brigitte Vollmar, Marc-André Weber, Bernd J. Krause, Timo Kirschstein, Katrin Porath, Nadja Engel, Stefan Teipel, Alexander Radbruch, Katerina Deike, Angela Kuhla

**Affiliations:** 1 Rudolf-Zenker-Institute for Experimental Surgery, Rostock University Medical Centre, Schillingalle 69a, 18057 Rostock, Germany; 2 Department of Psychosomatic Medicine and Psychotherapy, Rostock University Medical Centre, 18147 Rostock, Germany; 3 Centre for Transdisciplinary Neurosciences Rostock (CTNR), Rostock University Medical Centre, 18147 Rostock, Germany; 4 Core Facility Multimodal Small Animal Imaging, Rostock University Medical Centre, 18057 Rostock, Germany; 5 Institute and Policlinic of Radiology, Pediatric Radiology and Neuroradiology, Rostock University Medical Centre, 18057 Rostock, Germany; 6 Department of Nuclear Medicine, Rostock University Medical Center, 18057 Rostock, Germany; 7 Oscar Langendorff Institute of Physiology, University of Rostock, Rostock, Germany; 8 Department of Oral, Maxillofacial and Plastic Surgery, Rostock University Medical Center, Rostock, Germany; 9 German Center for Neurodegenerative Diseases (DZNE), Rostock and Greifswald, 18147 Rostock, Germany; 10 Clinic for Neuroradiology, University Clinic Bonn, 53127 Bonn, Germany; 11 German Center for Neurodegenerative Diseases (DZNE) Bonn, 53127 Bonn, Germany

**Keywords:** Alzheimer´s disease, Gadolinium-based contrast agent, Magnetic resonance imaging, Translational model, Brain Clearance, Cerebrospinal fluid

## Abstract

**Background:**

Brain clearance pathways via the interstitial fluid and cerebrospinal fluid are essential for maintaining homeostasis and removing metabolic waste. Disruptions in these processes are linked to neurodegenerative diseases, particularly Alzheimer’s Disease (AD), where an imbalance between amyloid β (Aβ) production and clearance leads to cerebral amyloidosis. Although AD research continues to rely heavily on murine models, including APPswe/PS1dE9 mice, it remains unclear to what extent these models replicate the deficits in brain clearance observed in human AD.

**Methods:**

We examined AD pathology in transgenic APPswe/PS1dE9 (tg) mice and wildtype (wt) littermates at six, nine and twelve months using immunohistochemistry for Aβ plaques (6E10^+^) and activated microglia (Iba1^+^). To assess integrative parameters contributing to brain clearance, we applied two complementary approaches. First, we performed daytime-dependent intrahippocampal injections of a fluorescent tracer in six-month-old tg and wt mice, followed by histological analysis of tracer dispersion 1 h and 3 h after injection. Second, we present a novel, longitudinal and non-invasive approach utilizing washout kinetics of intravenously administered gadolinium-based contrast agents (GBCA) as an integrative proxy to assess brain clearance dynamics. In this novel approach DOTAREM^®^ was administered intravenously and imaging was done with anesthetized tg and wt mice at nine and twelve months of age. Integrity of the blood-brain-barrier was further evaluated via western blot analysis of occludin protein expression.

**Results:**

Despite tg mice displayed characteristic AD-like phenotype, with significant, age-dependent Aβ plaque deposition and neuroinflammation no significant differences in the integrative GBCA washout kinetics were detected between wt and tg mice, suggesting that no major deficits in the combined clearance pathways are present. However, contrast-agent uptake showed a general age-dependent increase consistent with aging-related changes known from human studies.

**Conclusion:**

Our findings indicate that the APPswe/PS1dE9 mouse model does not exhibit evidence of major integrative brain clearance impairment, despite this being a hallmark of sporadic AD in humans. These results suggest limitations of this model for studying clearance-related mechanisms of late-onset AD, which should be acknowledged when planning future studies using this murine model.

**Supplementary Information:**

The online version contains supplementary material available at https://doi.org/10.1186/s12987-026-00872-9.

## Background

In addition to neurons and glial cells, the brain consists of vasculature and fluid-filled spaces (smaller extracellular/interstitial spaces and larger ventricular areas) which altogether are accountable for approximately 15–20% of the total brain volume [1]. One fluid is interstitial fluid (ISF) that contains different ions, as well as a variety of gaseous and organic molecules, building a microenvironment in the extracellular space of neurons and glial cells [1–3]. The second is cerebrospinal fluid (CSF) filling the subarachnoid space and ventricle system. Both compartments are in close communication with each other [4, 5]. Physiological movement and interchange of ISF and CSF in the brain serves two pivotal functions: (i) distribution of nutrients and neuromodulators, and (ii) elimination of possibly toxic metabolic waste products [6–8]. Due to the high metabolic rate of neurons [9], the clearance of these waste products is of utmost importance to maintain homeostasis and regular neuronal function. Disruptions in these processes of brain clearance were identified as pathogenic factors in different neurodegenerative diseases with protein aggregations, including Alzheimer’s disease (AD) which is characterized by the accumulation of amyloid β (Aβ) 42 [10].

The recent revision of the amyloid cascade hypothesis of AD considers a range of underlying mechanisms, but still proposes that cerebral amyloidosis is the main driver of AD pathogenesis [11–13]. This amyloidosis is thought to be caused by an imbalance of production and clearance of amyloid peptides, like Aβ42. In familial forms of early-onset AD due to variant mutations e.g., in the amyloid precursor protein (APP) or in presenilin (PS) genes, overproduction of insoluble Aβ species is considered the primary pathogenic event [14]. However, familial forms of early-onset AD are estimated to represent less than 1% of AD cases [15] and in the much more common form of sporadic late-onset AD it is discussed that disruptions in brain clearance function may have a more pivotal role as pathogenic driver of disease progression [16–18].

To measure brain clearance function there is already a variety of literature regarding the application of gadolinium-based contrast agents (GBCA)-enhanced magnetic resonance imaging (MRI) measurement. These include clinical [19–22] as well as preclinical studies with rats [23, 24]. Nevertheless, sparse research applying GBCA-enhanced MRI in mice use invasive intracisternal injections [25, 26]. This leads to a limited translational potential although mice are predominantly used models in basic research and therapeutic development in AD.

For that reason, we present a novel method of intravenously administered GBCA-enhanced MRI analysis in anesthetized transgenic APPswe/PS1dE9 (tg) mice, a broadly used AD model, and their non-transgenic/wildtype (wt) littermates. We compared this method with an ex vivo method of fluorescence dye dispersion as proxy of brain clearance. Using both methods we aimed to test the hypothesis that tg mice showed impaired clearance compared to wt controls, measured as a delayed and decreased dye dispersion and a prolonged washout phase in MRI, respectively. In perspective, the MRI-based method may offer versatile applications in basic research with potential clinical relevance. The usage of gadoteric acid (DOTAREM^®^) with eligible use in human brain MRI and the minimally invasive intravenous application underline the translational potential of the herein presented method. We hypothesized that the APPswe/PS1dE9 model exhibits age-dependent impairments in ventricular and parenchymal brain clearance, thereby partially recapitulating clearance deficits described in sporadic late-onset Alzheimer’s disease.

## Methods

### Animals

All animal experimental work was carried out with permission of the local Animal Research Committee (Landesamt für Landwirtschaft, Lebensmittelsicherheit und Fischerei (LALLF)) of the state Mecklenburg-Western Pomerania (LALLF M-V/TSD/7221.3-2-009/20) and all animals received human care according to the EU Directive 2010/63/EU.

In this experimental approach we used female tg mice of the AD model APPswe/PS1dE9 on a C57BL6JxCH3 background and their wt littermates at the age of six, nine and twelve months. Mice were bred in the core facility for laboratory animals of the Rostock University Medical Centre. Mice were kept in groups of three to five in standard cages with a defined room temperature (21 ± 3 °C) and a 12/12°hour day-night-cycle (lights on from 6°am to 6°pm). All mice had *ad libitum* access to food and water. In compliance with our own previous and ongoing investigations, female mice were used for comparability between different studies.

At the end of the respective experimental time, mice were euthanized under deep anaesthesia with isoflurane (5% Baxter, Unterschleißheim, Germany) with cervical dislocation.

### Immunohistological examination of Aβ-pathology and neuroinflammation

Brain tissue from tg and wt mice at six, nine and twelve months of age that were neither used in the fluorescent tracer experiments nor in the GBCA-imaging experiments was fixed in 4% phosphate-buffered formalin, embedded in paraffin and sliced sagittally starting at the midline into 4 μm-thin sections. Sections were put on X-tra Adhesive Precleaned Micro Slides (Leica, Wetzlar, Germany) and exposed to mouse monoclonal anti-Aβ antibody (clone 6E10; 1:1000, BioLegend, San Diego, CA, USA) and goat polyclonal anti-Iba1 antibody (1:1000, Abcam, Berlin, Germany), as previously described by our group [27]. Slices were approximately 4–24 μm apart; therefore, analysed regions of interest of the frontal cortex and hippocampus were comparable between the two stainings and between different animals. DAB chromogen Universal LSAB^®^ kits (System-HRP; DakoCytomation, Dako, Jena, Germany) were used for development according to the manufacturer’s instructions. Sections were counterstained with hemalaun (Merck, Darmstadt, Germany), and images were acquired on microscope type BX51 with a Color View Soft Imaging System and the corresponding software cellSens Standard 1.14 (all from Olympus, Hamburg, Germany).

Quantification of Aβ-pathology (Supplementary Fig. 1) was performed as (i) number of individual 6E10^+^ plaques per mm² of the tissue (independently of the plaque size); (ii) average size of individual 6E10^+^ plaques (in µm²) per sample (independently of the total number of plaques) and (iii) total combined area (in µm²) of tissue covered by 6E10^+^ plaques per visual field.

### Fluorescent tracer experiments

As assessment for brain clearance, dispersion of a fluorescent dye was measured after stereotactic injection into the brain parenchyma in a daytime dependent manner. Of each genotype (tg or wt) 12 mice at an age of six months were blindly divided into four groups: group (A) 6 am with 1 h dispersion time; group (B) 6 am with 3 h dispersion time; group (C) 6 pm with 1 h dispersion time; and group (D) 6 pm with 3 h dispersion time, with each group containing 3 mice.

#### Stereotactic dye injection

Starting two days prior to the injection surgery, mice received pain medication with Metamizol (Novaminsulfon-ratiopharm^®^, ratiopharm GmbH, Ulm, Germany) periorally with their drinking water (3 mg/ml), to obtain preemptively sufficient blood concentrations of the analgesic. Before surgery, mice received a weight-adapted intraperitoneal injection of a mixture of ketamine (98 mg/kg bodyweight, medistar, Ascheberg, Germany) and xylazine (6.5 mg/kg bodyweight, Bayer, Leverkusen, Germany). Under anesthesia, head fur was shaved, and the mouse was fixed in the stereotactic frame. The skin was incised with a scalpel and the skull was carefully dissected free. The skull was then opened with a small trepanation (diameter < 2 mm) and a fine needle (Hamilton 701 N, Hamilton Company, Reno, Nevada, USA) was used to inject 1 µl of fluorescent dye (Kaleidoscope™, Biorad, Feldkirchen, Germany) into the hippocampal area of the mouse brain at the following coordinates relative to bregma: -3.0 mm AP, -3.0 mm ML, -3.0 mm SI (from skull). To minimize brain damage due to injection pressure, the total volume was injected slowly over a time course of 10 min. After injection, the needle remained in the brain for another 5 min to minimize reflux. After needle withdrawal, the skin incision was closed with tissue adhesive (Surgibond, SMI AG, St. Vith, Belgium). Surgeries were performed in a daytime dependent manner with group A and B injected at 6 am (dusk for the mice, begin of the sleep phase) and group C and D injected 6 pm (dawn for the mice, begin of the wake phase). After the surgical procedure, mice were allowed to recover for 1 h (group A and C) or 3 h (group B and D) before cervical dislocation under deep anesthesia and immediate brain removal for histological analysis.

#### Histological analysis

Brain tissue was fixed in 4% formafix (Grimm med. Logistik GmbH, Torgelow, Germany) and embedded in paraffin. Both hemispheres were sliced transversally each 210 μm into three 4 μm thin sections and slices were put on Polysine^®^ slides (Menzel, Braunschweig, Germany). This led us to approx. 12–18 different transversal layers of each hemisphere. All transversal slices were microscopically assessed. Representative images are shown in the Supplementary Fig. 2. Images were acquired on Zeiss Axio Imager.M2 microscope with Zeiss AxioCam MRc5 and the corresponding Zeiss Zen 2 Pro software (all from Carl Zeiss, Oberkochen, Germany). Images were manually corrected for artifacts (e.g., removal of folds in the tissue before subsequent analysis). As all injections were administered to the right hemispheres, the left hemispheres could be used as internal controls for threshold corrections. Tissue dispersion of the fluorescent dye was quantified semi-automatically with ImageJ 1.47 v (Supplementary Code 1) and calculated as percentage of area above fluorescent threshold/total brain tissue area.

### GBCA-imaging experiments

As approach for reduction and refinement in accordance with the 3R principles of animal research, instead of measurement of brain clearance efficacy by highly invasive tracer injections as endpoint measurement (described in 2.3.), we now describe a novel method using washout kinetics of intravenously administered GBCA as an integrative proxy to assess brain clearance dynamics. This method minimized invasiveness, enhanced overall animal-welfare and allowed us now longitudinal measurements of the same individual animals over time.

#### Magnetic resonance imaging procedure

Imaging sessions were performed longitudinally at nine and twelve months of age. During the whole procedure at each session, mice were anesthetized using 1.5–2.5% isoflurane (Baxter, Unterschleißheim, Germany) with oxygen (Air Liquide, Hamburg, Germany). Mice were placed in a prone position in a 7 Tesla small animal MRI-scanner (Bruker BioSpin GmbH, Ettlingen, Germany) equipped with a ^1^H cryogenic, two elements, transmit/receive coil array. Mice were able to breathe spontaneously. The heartrate as well as the respiration of the mice was monitored during the imaging session. The core body temperature of the animals was maintained at 37 °C via a temperature-controlled heating pad.

The scan protocol consisted of a t1w rapid acquisition with relaxation enhancement (RARE) sequence in all mice (9 wt and 9 tg). In 5 wt and 5 tg mice this sequence was additionally alternated with a highly t2w inversion recovery RARE fluid attenuation inversion recovery (FLAIR) Sequence: t1w RARE transversal: TE/TR: 16ms/1160ms, Rare factor: 8, FoV: (20 × 21) mm, image matrix: 234 × 246, resolution: (85 × 85) µm, slice thickness 500 μm, slices 16, TA: 2:40 min: s t2w RARE inversion recovery transversal: TE/TR/TI: 37ms/10000ms/1800ms, Echo spacing: 12.333 ms, Rare factor 8, FoV: (20 × 21) mm, image matrix: 234 × 246, resolution: (85 × 85) µm, slice thickness 500 μm, slices 16, TA: 3:50 min: s. For better B_0_-homogeniety, a B_0_-map with subsequent map-shim procedure on the brain was carried out.

After baseline scans with the two sequences, mice received intravenously administered gadoteric acid (DOTAREM^®^ 0.5 mmol/ml, Guerbet, Sulzbach, Germany). The contrast agent DOTAREM was administered as a bolus at a volume of 0.0025 ml/g of bodyweight via tail vein catheter. Dosage was calculated as the animal equivalent dose of the human standard dose of 0.1 mmol/kg (manufacturer’s information and recommendation for MRI of the human brain and spinal cord) according to body surface area normalization [28].

Contrast enhanced t1w alone or alternated with postcontrast FLAIR (t2w) sequences were carried out for a maximum of 182 min after administration (each single measurement took approx. 2–5 min). This time span was chosen to minimize the risks associated with prolonged anaesthesia and to comply with animal welfare regulations.

#### VOI Characterization

In t2w imaging data Volumes of interest (VOIs) in both lateral ventricles as well as the 3rd and 4th ventricle in axial brain slices  -2.2 mm — -2.4 mm dorsal to the Bregma were manually mapped using PMOD software (version 3.7; PMOD Technologies LLC, Zürich, Switzerland).

Additionally, in t1w imaging data bilateral VOIs in frontal cortex and hippocampus were manually mapped using the same PMOD software (version 3.7; PMOD Technologies LLC, Zürich, Switzerland).

#### Data analyses

Data analysis was performed using PMOD software (version 3.7; PMOD Technologies LLC, Zürich, Switzerland). With the built-in function ‘calculate selected VOI statistics’ mean pixel values (arbitrary units) were calculated as a measure for brightness in the selected VOI. The brightness thereby served as an indirect measurement for GBCA concentration in tissue. Based on these values, intensity-time-curves showing the relative change of mean pixel values of the VOIs as a percentage starting from baseline level (= 0%) were created.

Further analysis of these intensity-time-curves was performed with the software IgorPro V6.37 (WaveMetrics, Portland, OR, USA). The signal intensity increase after contrast agent injection and the subsequent decrease of the signal during the clearance-phase were described with a single-model-function consisting of an exponential convoluted with another exponential (build in function in IgorPro V6.37). An example of the fitted model is presented as Supplementary Fig. 3. We choose this model based on the assumption that within the ventricles, the contrast agent uptake and clearance are to a large extent diffusion-driven processes, which typically can be well described within an exponential approach [29, 30]. Fitting all experimental data with that model function yielded the exponential decay-constants “c1” and “c2” for the rising and the falling part of each intensity-time-curve, respectively. Since the decay-constants are independent of the signal intensity, they can be used directly for animal-to-animal comparison without any need for further data normalization.

### Western Blot

Following final GBCA-imaging measurements in twelve-month-old mice, the harvested brain tissue was processed for protein isolation. For this purpose, brain tissue was homogenized in lysis buffer (10 mM Tris pH 7.5, 10 mM NaCl, 0.1 mM EDTA, 0.5% Triton-X100, 0.02% NaN_3_ and 0.2 mM PMSF, protease inhibitor cocktail), incubated for 30 min on ice and centrifuged for 10 min at 4 °C and 10.000 x g. Protein contents were assayed by bicinchoninic acid method (Pierce Biotechnology Inc.,Thermo Fisher Scientific, Waltham, MA, USA) with 2.5% BSA (Pierce Biotechnology Inc.,Thermo Fisher Scientific, Waltham, MA, USA) as standard. On a 10% SDS gel 10 µg protein was separated and transferred to a polyvinyldifluoride membrane (Immobilon-P; Millipore, Eschborn, Germany). After blockade with 2.5% BSA (Santa Cruz Biotechnology, Santa Cruz, CA, USA), membranes were incubated overnight at 4 °C with a rabbit polyclonal anti-occludin (1:1.000, invitrogen, Darmstadt, Germany). Afterwards, a secondary peroxidase-linked anti-rabbit antibody (1:10.000; Cell Signaling Technology, Cambridge, UK) was applied. Protein expression was visualized by means of luminol-enhanced chemiluminescence (ECL plus; Amersham Pharmacia Biotech, Freiburg, Germany) and digitalized with ChemiDoc™ XRS System (Bio-Rad Laboratories, Feldkirchen, Germany). Signals were assessed densitometrically (Quantity One; Bio-Rad Laboratories, Munich, Germany) and normalized to the GAPDH signals (mouse monoclonal anti-β-GAPDH antibody; 1:20.000; Millipore, Eschborn, Germany, followed by second antibody anti-mouse, 1:40.000, Sigma Aldrich Corp., St. Louis, MO, USA).

### Statistics

Data visualization and statistical analysis was performed using GraphPad Prism 8.0.1 (GraphPad Software Inc., San Diego, CA, USA). Data are presented as mean ± standard deviation if not stated otherwise, and statistical significance was set at *p* < 0.05. Normal distribution of data was tested using Shapiro-Wilk test. For further details, please see figure legends.

## Results

### Characterization of AD phenotype in mice

To characterize AD disease progression, brains from tg and wt mice at six, nine and twelve months of age (that were neither used in the fluorescent tracer experiments nor in the GBCA-imaging experiments), were analysed for Aβ-deposition and neuroinflammation.

#### Aβ-deposition

**Fig. 1:**
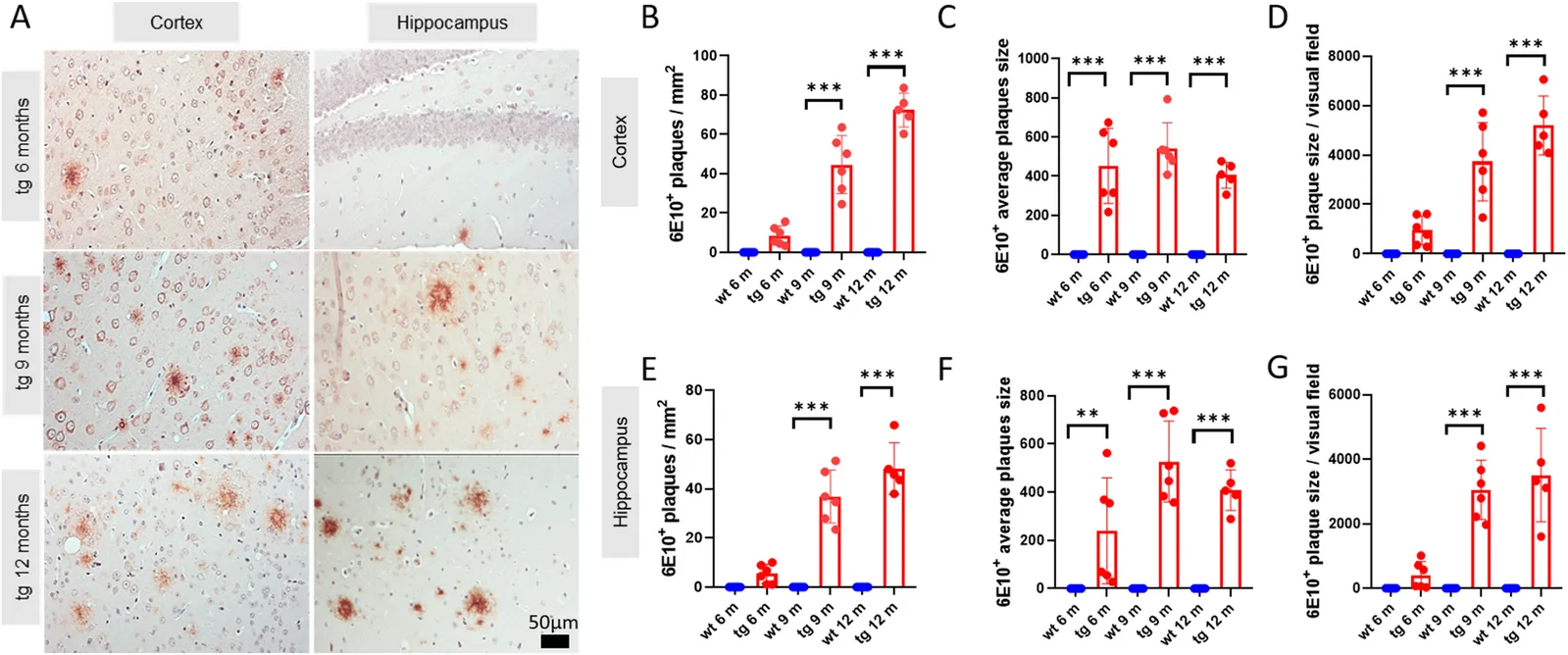
Immunohistological analysis of amyloid β plaques (6E10^+^) in brains of APPswe/PS1dE9 (tg) mice and their wildtype (wt) littermates at ages of 6, 9 and 12 months, respectively. (**A**) Representative images of 6E10+ tissue in cortex (left) and hippocampus (right) of tg mice at 6 (top), 9 (middle) and 12 (bottom) months. Scale bar 50 μm. Corresponding images of wt mice are presented as Supplementary Fig. 1. (**B**) Analysis of total numbers of 6E10+ plaques per mm² in cortex. (**C**) Analysis of average 6E10+ plaques size in cortex. (**D**) Analysis of total area of 6E10+ plaques per field of view in cortex. (**E**) Analysis of total numbers of 6E10+ plaques per mm² in hippocampus. (**F**) Analysis of average 6E10+ plaques size in hippocampus. (**G**) Analysis of total area of 6E10+ plaques per field of view in hippocampus. n = 6 mice per group, except tg 12 months n = 5. Data presented as individual data points with mean ± SD. No 6E10+ measurements were detected in wildtype mice (n.d.). Data was normally distributed (Shapiro-Wilk test). 2-Way ANOVA followed by Sidak’s correction for multiple comparisons, ** p < 0.01; *** p < 0.001

First, we examined the deposition of Aβ-plaques (6E10^+^) in the brains, with focus on cortex and hippocampus (Fig. 1A; Supplementary Fig. 4). A 2-way ANOVA revealed both, a significant effect of genotype and age for the total number of 6E10^+^ plaques in cortex (genotype: F_(1, 29)_ = 210.0, *p* < 0.0001; age: F_(2, 29)_ = 37.9, *p* < 0.0001) and hippocampus (genotype: F_(1, 29)_ = 295.3, *p* < 0.0001; age: F_(2, 29)_ = 56.9, *p* < 0.0001) (Fig. 1B and E). However, quantification of average plaque size of 6E10^+^ plaques was not as uniformly as expected and did not seem to be age related in cortex (F_(2, 29)_ = 1.39, *p* = 0.2653) but in hippocampus (F_(2, 29)_ = 4.4, *p* = 0.0210). A statistically significant effect of genotype was found in both cortex (F_(1, 29)_ = 190.5, *p* < 0.0001) and hippocampus (F_(1, 29)_ = 94.5, *p* < 0.0001) (Fig. 1C and F). Furthermore, a significant effect of genotype and age was observed for the quantification of total 6E10^+^ plaque area per visual field in cortex (genotype: F_(1, 29)_ = 136.7, *p* < 0.0001; age: F_(2, 29)_ = 19.4, *p* < 0.0001) and hippocampus (genotype: F_(1, 29)_ = 101.5, *p* < 0.0001; age: F_(2, 29)_ = 17.7, *p* < 0.0001) (Fig. 1D and G).

#### Neuroinflammation

**Fig. 2:**
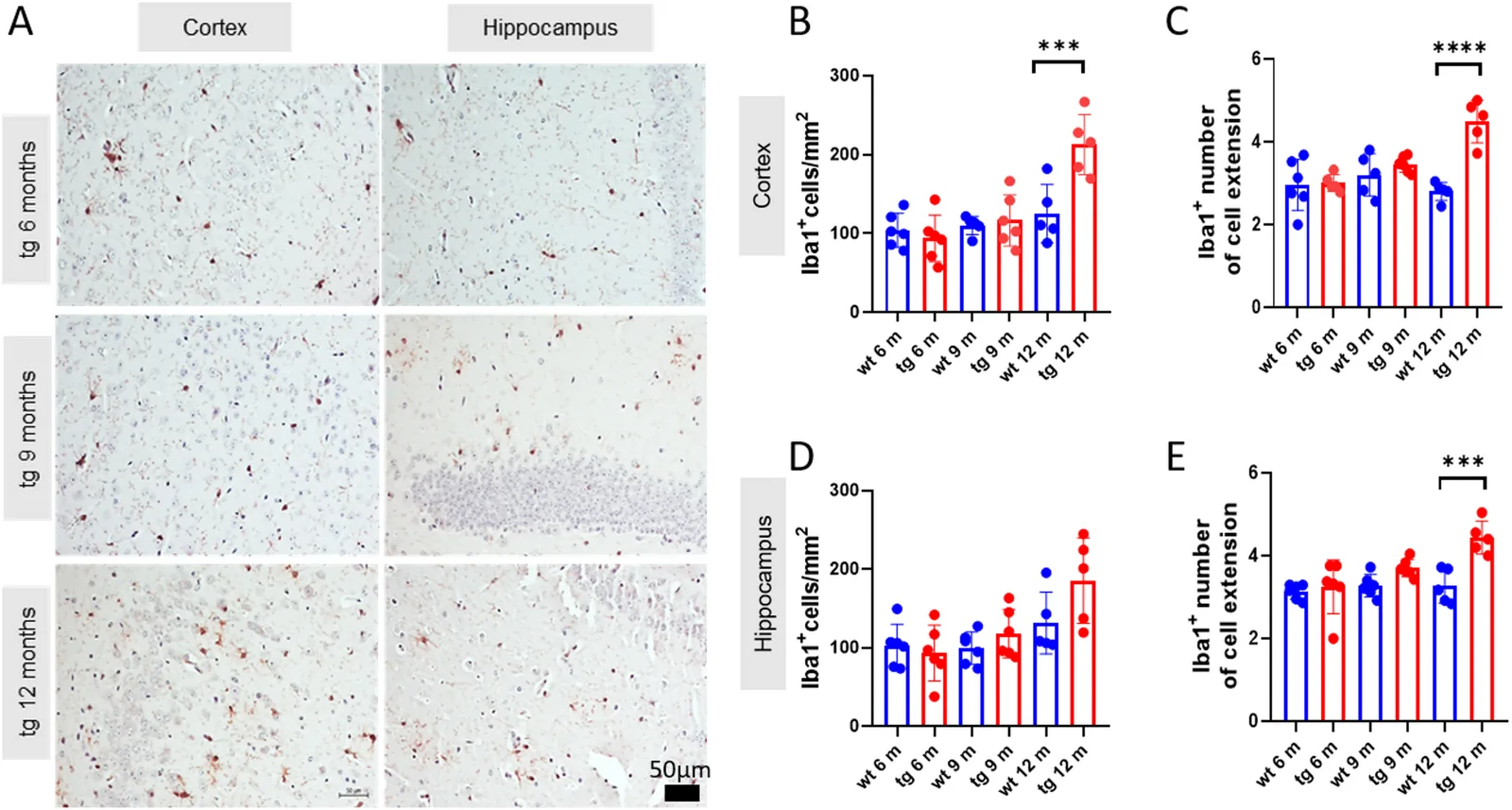
Immunohistological analysis of activated microglia (Iba1^+^) in brains of APPswe/PS1dE9 (tg) mice and their wildtype (wt) littermates at ages of 6, 9 and 12 months, respectively. (**A**) Representative images of Iba1+ cells in cortex (left) and hippocampus (right) of tg mice at 6 (top), 9 (middle) and 12 (bottom) months. Corresponding images of wt mice are presented as Supplementary Figure 2. (**B**) Analysis of total numbers of Iba1+ cells per mm² in cortex. (**C**) Analysis of average number of cell extensions per Iba1+ cell in cortex. (**D**) Analysis of total numbers of Iba1+ cells per mm² in hippocampus. (**E**) Analysis of average number of cell extensions per Iba1+ cell in hippocampus. n = 6 mice wt 6 months, n = 5 mice wt 9 and 12 months, n = 6 mice tg 6 and 9 months, n = 5 mice tg 12 months. Data presented as individual data points with mean±SD. Data was normally distributed (Shapiro-Wilk test). 2-Way ANOVA followed by Sidak's correction for multiple comparisons, *** p < 0.001; **** p < 0.0001

Using the same samples, we examined neuroinflammation by quantification of activated microglia (Iba1^+^) in the brains, with focus on cortex and hippocampus (Fig. 2A; Supplementary Fig. 5).

2-way ANOVA revealed both, a significant effect of genotype and age for the total number of Iba1^+^ cells in cortex (genotype: F_(1, 27)_ = 7.3, *p* = 0.0119; age: F_(2, 27)_ = 16.5, *p* < 0.0001) and for age in the hippocampus (age: F_(2,28)_ = 8.9, *p* = 0.0010) (Fig. 2B and D). After Sidak’s correction for multiple comparisons, differences were significant at an age of 12 months in the cortex (*p* = 0.0002). The same was observed for the quantification of the average number of cell extensions per Iba1^+^ cell in cortex (genotype: F_(1, 27)_ = 19.8, *p* = 0.0001; age: F_(2, 27)_ = 6.5, *p* = 0.0050) and hippocampus (genotype: F_(1, 27)_ = 18.3, *p* = 0.0002; age: F_(2, 27)_ = 8.0, *p* = 0.0017) (Fig. 2C and E). After Sidak’s correction for multiple comparisons, differences between wt and tg were significant at an age of 12 months (cortex: *p* < 0.0001, hippocampus: *p* = 0.0001). Confirmation of a robust AD-like phenotype, characterized by both Aβ-plaque formation and neuroinflammation, was a prerequisite for the planned experiments on brain clearance. The detection of tissue deposition of Aβ-plaques phenotypic for AD starting at 6 months of age in tg mice led us to perform the fluorescent tracer experiments in mice at an age of 6 months.

### Fluorescent tracer experiments

**Fig. 3:**
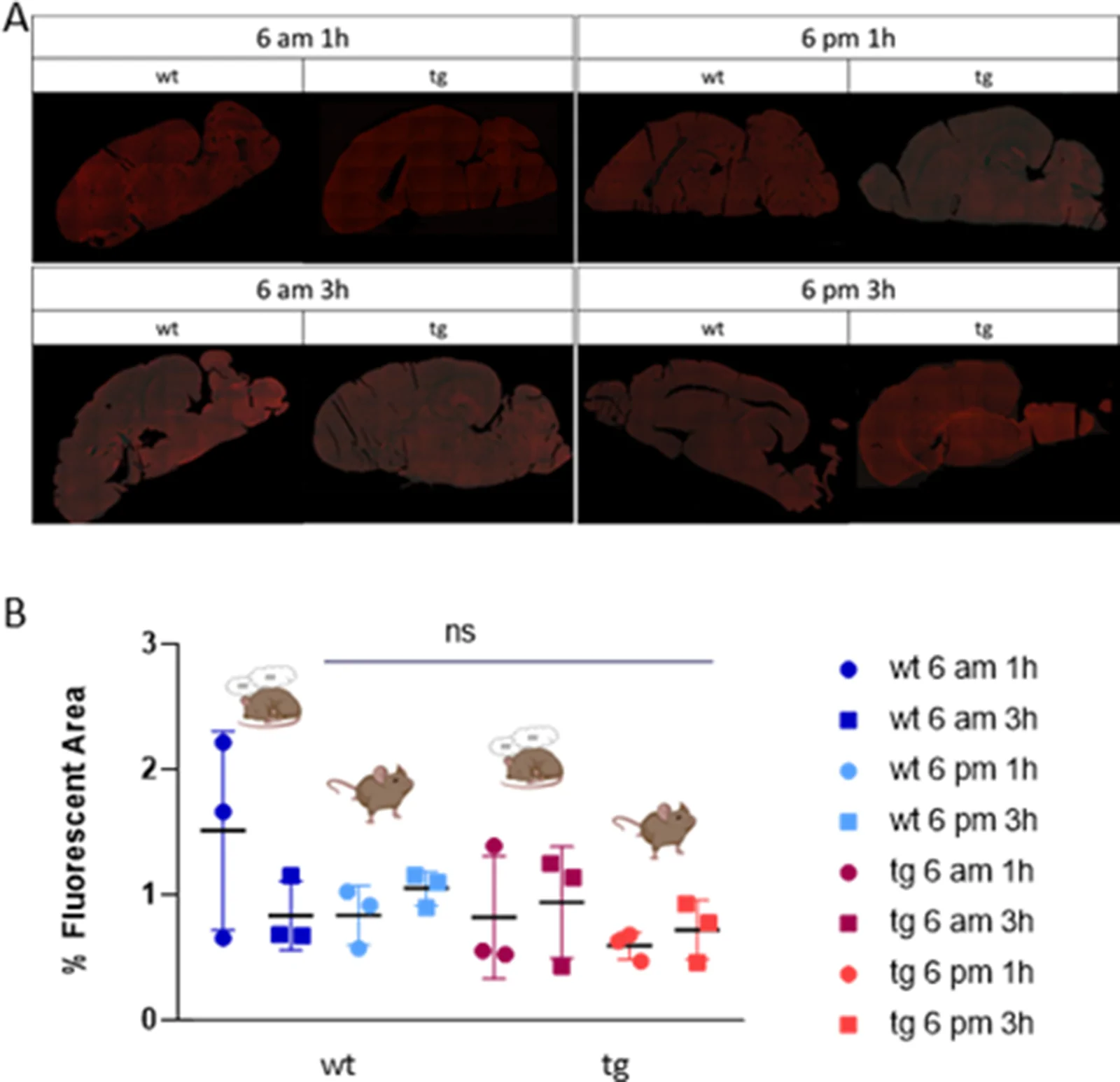
Quantification of daytime-dependent fluorescent tracer dispersions in brains of APPswe/PS1dE9 (tg) mice and their wildtype (wt) littermates as a measure for brain clearance. (**A**) Representative images of fluorescence in an axial sliced brain section (approx. 2.5 - 3 mm dorsal to the bregma) of wt and tg mice at the different groups (6 am vs. 6 pm, 1 h or 3 h post-injection; n = 3 per group). (**B**) Dye dispersion as % fluorescent area of the whole tissue sample. Individual data points represent the mean value of 12 - 18 brain sections of a single mouse, while the line indicates the group mean with error bars representing the standard deviation. Data was not normally distributed (Shapiro-Wilk test). Kruskal-Wallis test revealed no statistical significance between the different groups. Created in BioRender. Kuhla, A. (https://BioRender.com/9wmf8zf) is licensed under CC BY 4.0)

Following the injection of a fluorescent tracer in wt and tg mice at the age of six months either at 6 am or 6 pm, tracer dispersion after 1 and 3 h was measured in histologic samples of the mouse brain (Fig. 3A). Dispersion was measured as fluorescence intensity in transversal brain slices and the reported value is the mean per animal throughout all sections of the hemisphere with the injection. We were not able to find statistically significant changes between wt and tg mice at the different endpoint measurements (Fig. 3B).

### GBCA-imaging experiments

As we did not previously see brain clearance impairment within the fluorescent tracer experiments at the age of six months, with manifest Aβ-plaque pathology but without significant signs of neuroinflammation, we used now mice at nine months of age and performed a second measurement in those same mice at twelve months of age. Based on the age-dependent progressive pathology shown in 3.1, GBCA-imaging experiments were performed at nine and twelve months to evaluate brain clearance specifically in the context of manifest amyloidosis and neuroinflammation.

Nearly all mice (8/9 tg and 9/9 wt) remained stable during the experimental time and survived the anesthesia without any further restrictions in their well-being. One tg mouse died during MRI measurements at twelve months.

#### T2w images

**Fig. 4:**
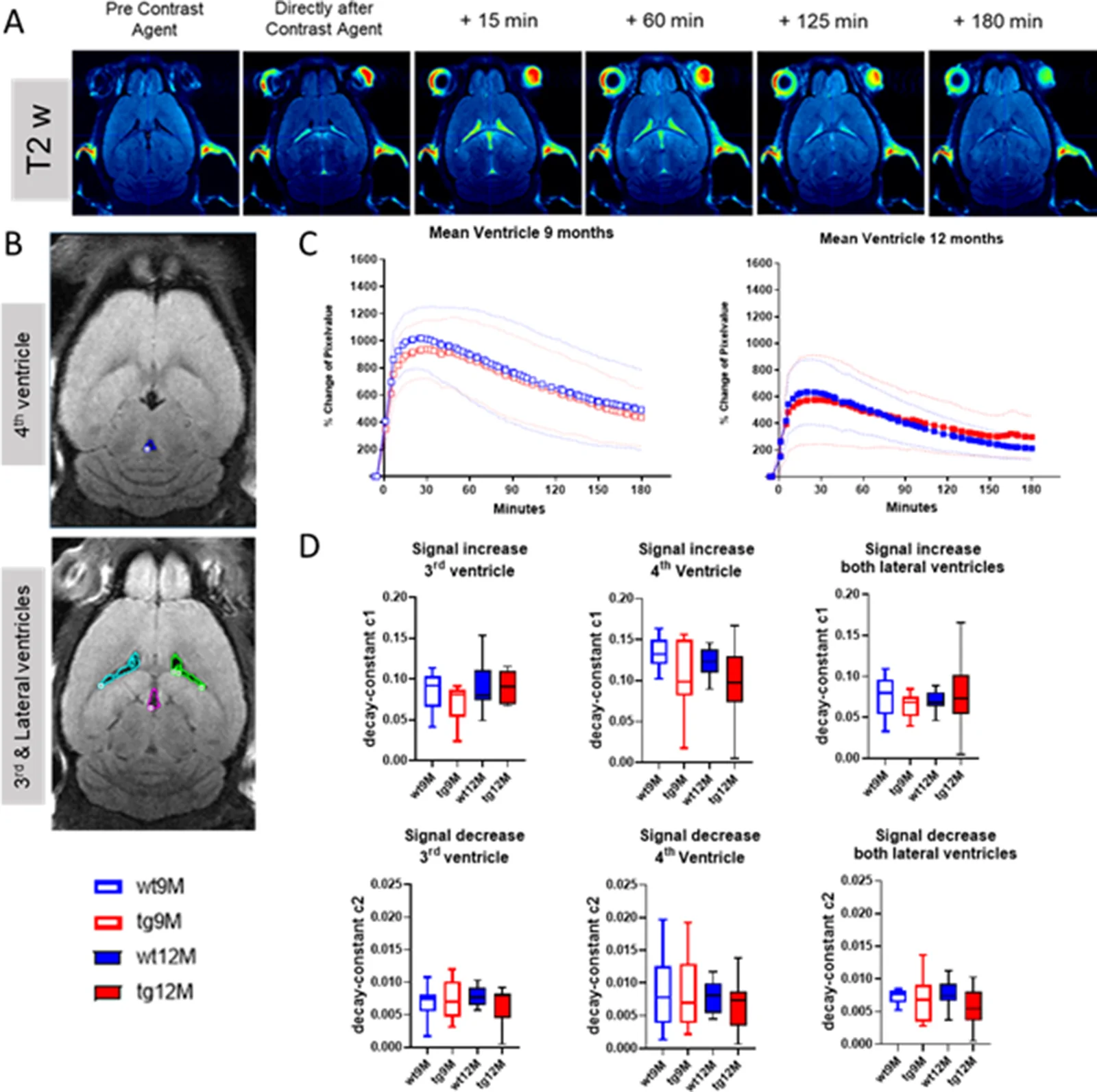
T2w GBCA-enhanced MRI measurements of mouse brains of APPswe/PS1dE9 (tg) mice and their wildtype (wt) littermates as a measure for ventricular brain clearance. (**A**) Representative timeline of t2w images before GBCA administration, directly after and at selected time points (15, 60 125 and 180 min after Injection). (**B**) Representative examples for manually mapped VOIs: 4th ventricle (blue, upper panel), both lateral ventricles (right: green, left: teal, lower panel) as well as the 3rd ventricle (pink, lower panel) in axial brain slices − 2.2 mm - -2.4 mm dorsal to the bregma. (**C**) Mean values of % change of pixel value (as a measurement for signal intensity in the respective volumes of interest (VOIs)) compared to baseline in wt (blue) and tg mice (red) at nine (left) and twelve months of age (right). Dotted lines in blue and red represent the standard deviation of the measurements. (**D**) Calculated decay-constant c1 of signal increase for 3rd, 4th and both lateral ventricles (i.e. invasion of GBCA into the ventricles, upper panel) and calculated decay-constant c2 of signal decrease for 3rd, 4th and both lateral ventricles (i.e. evasion of GBCA from the ventricles, lower panel). Data presented as boxplots, whiskers indicate minimal and maximal values. Data was normally distributed (Shapiro-Wilk test). Statistical analysis using repeated measures 2-way ANOVA or, if single values were missing after outlier correction, age matched mixed-effects model was applied (c1 3rd ventricle, c2 4th ventricle, c1 and c2 lateral ventricles)

In a first approach, we looked into t2w sequences to observe GBCA invasion and evasion (serving as a composite proxy for the various routes contributing to ventricular brain clearance) in both lateral ventricles, as well as in the 3rd and 4th ventricles over a time span of 180 min (last measurement time point for t2w sequences, Fig. 4A). Pixel values as a measure for signal intensity in manually delineated VOIs (Fig. 4B) were measured and used to calculate percentage changes of pixel value from baseline (image pre contrast agent administration, 100%) over time in wt and tg mice at nine and twelve months of age (Fig. 4C, data presented as mean of all ventricles for a better overview; data of distinct ventricles presented as Supplementary Fig. 6). The signal intensity change over time curves were used to calculate decay-constants c1 and c2 for signal increase and signal decrease to independently compare them between different ages and genotypes in both lateral ventricles, the 3rd and 4th ventricle (Fig. 4D). Repeated measures 2-way ANOVA revealed no significant effects of either age or genotype in 3rd ventricle (genotype: F_(1, 16)_ = 0.3182, *p* = 0.5805; age: F_(1, 16)_ = 0.0026, *p* = 0.9601), 4th ventricle (genotype: F_(1, 31)_ = 0.2774, *p* = 0.6022; age: F_(1, 31)_ = 0.4883, *p* = 0.4899) and the lateral ventricles (genotype: F_(1, 16)_ = 1.779, *p* = 0.1985; age: F_(1, 15)_ = 0.0235, *p* = 0.8802).

#### T1w images

**Fig. 5:**
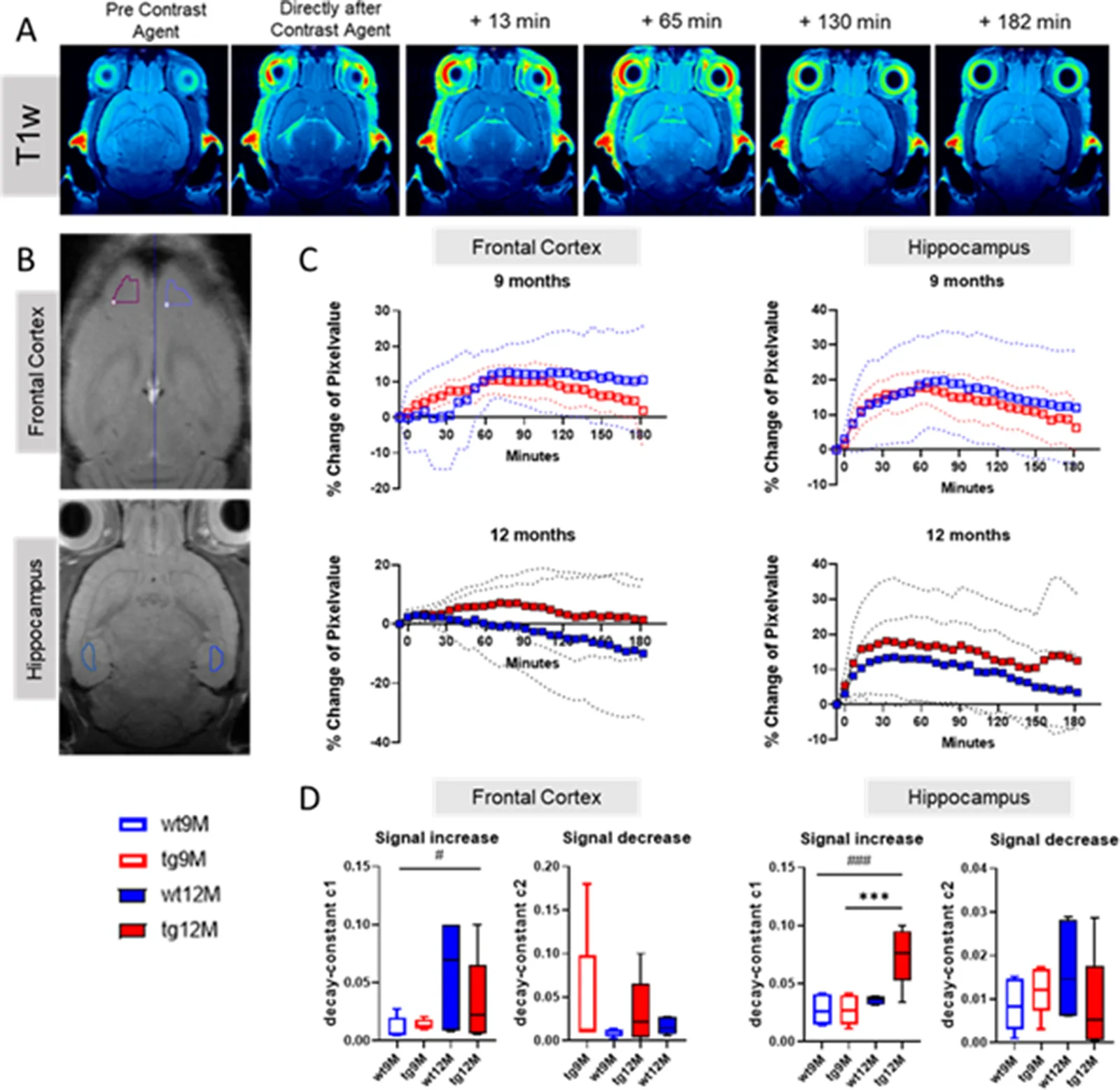
T1w GBCA-enhanced MRI measurements of mouse brains of APPswe/PS1dE9 (tg) mice and their wildtype (wt) littermates as a measure for parenchymal brain clearance. (**A**) Representative timeline of t1w images before GBCA administration, directly after and at selected time points (13, 65 130 and 182 min after Injection). (**B**) Representative examples for manually mapped VOIs of frontal cortex (left: violet and right: blue, upper panel) and hippocampus (left and right: blue, lower panel) in axial brain slices − 1.1 mm - -1.5 mm dorsal to the bregma. (**C**) Mean values of % change of pixelvalue (as a measurement for signal intensity in frontal cortex (left) and hippocampus (right) compared to baseline in wt (blue) and tg mice (red) at nine (upper panel) and 12 months of age (lower panel). (**D**) Calculated decay-constant c1 of signal increase for frontal cortex (left) and hippocampus (right) (i.e. invasion of GBCA into the parenchyma, upper panel) and calculated decay-constant c2 of signal decrease for frontal cortex (left) and hippocampus (right) (i.e. evasion of GBCA from the parenchyma, lower panel). Data presented as boxplots, whiskers indicate minimal and maximal values. Data was normally distributed (Shapiro-Wilk test). Statistical analysis using repeated measures 2-way ANOVA showed significant effects of age (# p < 0.05, ### p < 0.001), followed by Sidak’s correction for multiple comparisons *** p < 0.0001

In a second approach, we looked into t1w sequences to determine GBCA invasion and evasion (serving as a composite proxy for the various routes contributing to parenchymal brain clearance) into brain parenchyma over a time span of 182 min (last measurement time point for t1w sequences, Fig. 5A). Pixel values as a measure for signal intensity in manually delineated VOIs in frontal cortex and hippocampus (Fig. 5B) were measured and used to calculate percentage changes of pixel value from baseline (image pre contrast agent administration, 100%) over time in wt and tg mice at nine and twelve months of age (Fig. 5C). These signal intensity change-time curves were used to calculate decay-constants c1 and c2 for signal increase and signal decrease to independently compare between different ages and genotypes in frontal cortex (left) and hippocampus (right) (Fig. 5D). Repeated measures 2-way ANOVA revealed a significant effect of age in frontal cortex (F_(1, 8)_ = 6.5, *p* = 0.0344) and hippocampus (F_(1, 8)_ = 38.7, *p* = 0.0003) regarding signal increase. After Sidak’s correction for multiple comparisons, differences were significant in tg mice at the age of nine compared to twelve months in hippocampus (*p* = 0.0001). We observed no changes in heartbeat rate that could have artificially influenced the corresponding GBCA invasion measurements (Supplementary Fig. 7). However, no significant effects of age or genotype were observed regarding signal decrease as a measure for GBCA washout in frontal cortex (genotype: F_(1, 8)_ = 1.076, *p* = 0.3299; age: F_(1, 8)_ = 0.07253, *p* = 0.7945) and hippocampus (genotype: F_(1, 8)_ = 0.5154, *p* = 0.4933; age: F_(1, 8)_ = 0.1980, *p* = 0.6682).

### BBB Integrity

**Fig. 6:**
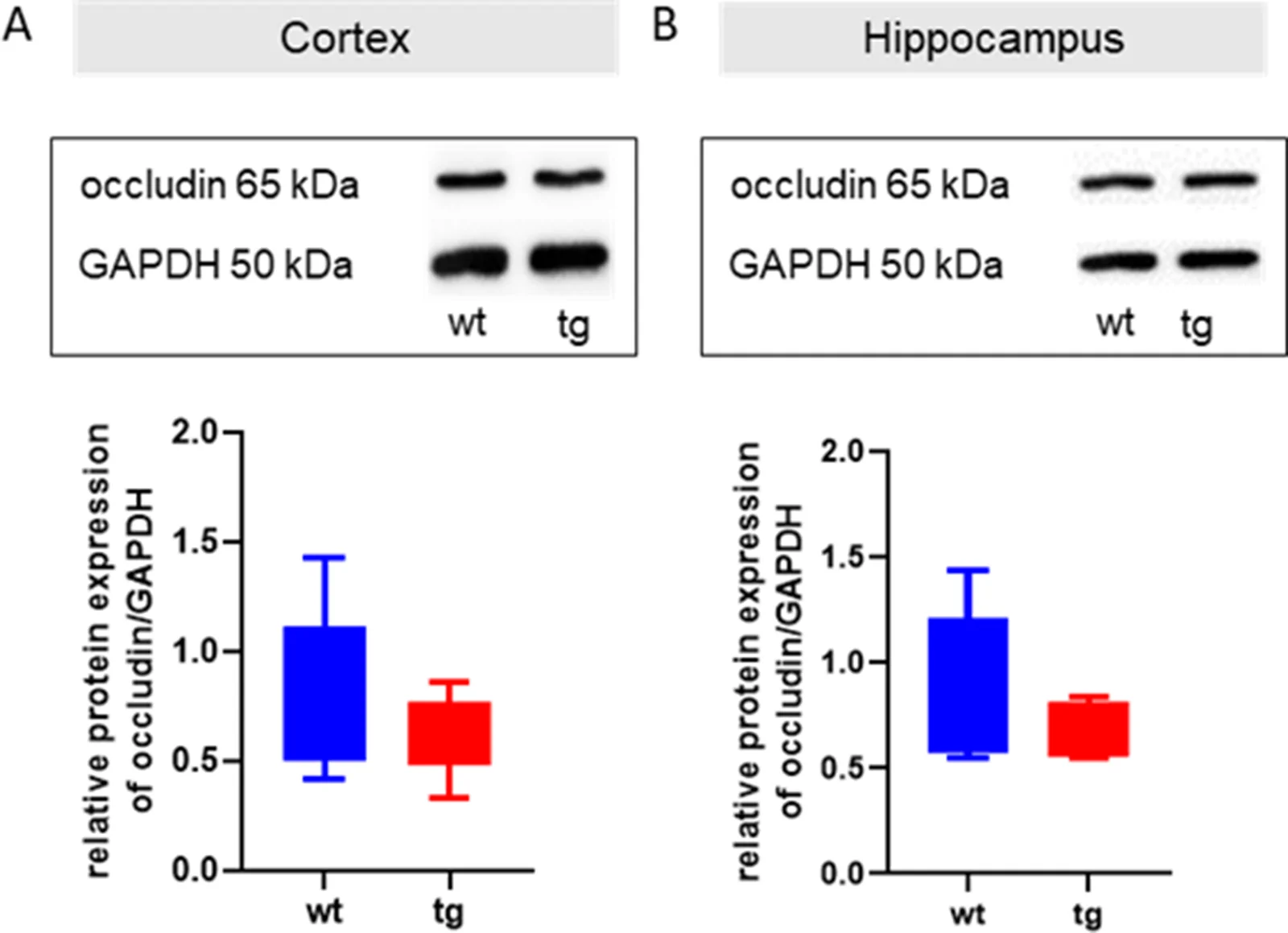
Western blot analysis of occludin protein expression in brains of APPswe/PS1dE9 (tg) mice and their wildtype (wt) littermates at the age of 12 months (*n* = 9 (tg), *n* = 7 (wt)). (**A**) Representative blots for cortex (left) and hippocampus (right) comparing occludin to the house-keeping protein GAPDH used for quantification presented in (**B**). Data presented as boxplots, whiskers indicate minimal and maximal values

As a possible explanation for the increased GBCA invasion into frontal cortex and parenchyma we investigated occludin protein expression as a measure for BBB integrity. This could only be performed in brain samples of the same mice when euthanized after the last in vivo imaging at the age of twelve months. Therefore, no comparison between nine and twelve months was possible. T-test showed no significant differences between wt and tg mice in cortex (t_(11)_ = 1.725, *p* = 0.1125) and hippocampus (t_(14)_ = 1.239, *p* = 0.2358), but tg mice showed generally a lower occludin protein expression (Fig. 6A and B, Supplementary Fig. 8).

## Discussion

Aging is an important factor influencing brain clearance mechanisms [31], and impairments in these processes are thought to contribute significantly to sporadic AD. However, it is not entirely clear to what extent brain clearance impairment is present in the APPswe/PS1dE9 model, a model based on Aβ overproduction, a key mechanism known from familial AD forms. Against our primary hypothesis within the experimental framework presented here, we detected no evidence of major integrative brain clearance impairment in either ventricular or parenchymal compartments of tg mice compared to their wt littermates despite manifest AD pathology. Therefore, we interpret that the AD-like phenotype observed in this specific AD model up to twelve months of age results primarily from the markedly increased Aβ production driven by the transgenic mutations.

In the study we undertook rigorous characterization of the AD phenotype in our tg mice up to an age of twelve months using histological and immunohistochemical methods, showing clear signs of Aβ-plaque formation and neuroinflammation. This detailed histological characterization shows essentially that tg mice used within the experimental approach had reached a robust state of pathology at the studied ages. We observed a significant age-dependent deposition of Aβ-plaques in both, hippocampus and cortex of tg mice compared to their wt littermates. The progression with age was observed in Aβ-plaque size and number, as quantified by 6E10^+^ immunohistochemical reactions. Additionally, both cortex and hippocampus showed substantial neuroinflammation, characterized by an increased number of Iba1^+^ cells, which mainly are activated microglia [32]. These results are in line with previously published results regarding this AD model [33–35] and starting point for our approaches to study brain clearance. As it is still a ‘chicken-or-egg’ causality dilemma in the pathogenesis of AD if amyloidosis or clearance impairment is the initiating factor, we aimed to investigate if clearance was altered at the earliest stage when amyloid pathology starts to become observable.

Based upon the demonstration of robust Aβ-pathology and neuroinflammation at six months, in our first approach we assessed brain clearance capacity in six months old tg mice and their wt littermates. Experimentally, we used histological assessment of the dispersion of a fluorescent dye injected into the hippocampal parenchyma. Experiments were carried out similar to intra-striatal, intra-cortical and intra-thalamic injections in C57BL/6 mice described by Iliff and colleagues [4]. However, the focus of the work was to qualitatively identify predominant parenchymal clearance routes along the internal cerebral and caudal rhinal veins using confocal microscopy [4]. In our own approach we used lower resolution microscopy for quantitative observation of dye dispersion after intra-hippocampal injection. Therefore, direct comparison of tracer dispersion results is not possible. Based on previous descriptions that brain clearance is influenced by circadian rhythm [36], we studied dye dispersion either at 6 am (dusk for the mice, begin of the sleep phase) or 6 pm (dawn for the mice, begin of the wake phase). Our obtained results showed no statistically significant difference in dye dispersion between wt and tg mice, regardless of daytime of dye injection or observed post-injection timepoint. Even though the total number of animals per group (*n* = 3) is very low, as this method is an endpoint measurement with very limited translational potential, we decided not to pursue this method any further and to carry out another experimental approach. A further limitation is that the fluorescent protein ladder used in this study does not match the molecular size and properties of Aβ, meaning that tracer dispersion may not fully reflect the transport and clearance behaviour of Aβ peptides and therefore provides only a coarse assessment of bulk parenchymal protein dispersion rather than a direct analysis of Aβ-specific transport or clearance mechanisms.

In contrast to previous approaches using intrathecal administered GBCAs for MRI-based investigations of brain clearance [23], we here present a novel method of intravenously administered GBCA-enhanced MRI analysis in anesthetized mice. This new approach has not only a higher translational potential of respective measurements -as it could possibly be adapted for measurements in human brain MRI- but also allow for investigation of GBCA invasion through blood-CSF-barrier [24, 37] and damaged BBB [37, 38]. Furthermore, this strategy enabled us to longitudinally observe ventricular and parenchymal brain clearance at the age of nine and twelve months, as there were no differences at the age of six months with the previously used dye dispersion measurements. T2w ventricular signal intensity analyses showed no genotype- or age-associated differences in GBCA invasion or clearance, suggesting a normal and stable CSF-mediated clearance in the observed mice. T1w measurements in brain parenchyma, i.e., frontal cortex and hippocampus, likewise revealed no significant effect regarding GBCA washout, suggesting a lack of notable changes in several pathways contributing to glymphatic clearance in the observed mice. The total measurement time in our experimental approach was 182 min and could not be elongated due to animal welfare reasons. Unfortunately, this time was not sufficient for complete washout of GBCA signal in ventricles and brain parenchyma and additional experiments using a similar imaging protocol but with elongated measurement time could add to more detailed insights on later stages during brain clearance but needs to be planned as final experiments, due to the increased stress and experimental burden for the mice, and cannot be conducted in a longitudinal approach.

A limitation of the current approach is that the GBCA washout (decay-constant c2) does not provide a direct measurement of glymphatic clearance, but rather acts as a composite proxy. The observed signal kinetics are influenced by a variety of factors, including BBB integrity and the complex interplay between interstitial fluid movement and CSF drainage. Therefore, we interpret the absence of significant differences in washout kinetics between tg and wt mice as a lack of major impairment in this integrative clearance marker, rather than a definitive statement on a single physiological brain clearance pathway.

Interestingly, t1w measurements revealed a statistically significant age effect on GBCA signal increase during the invasion into frontal cortex and hippocampal parenchyma. This reflects typical observations on increased BBB-permeability with age [39, 40] that is also reflected in various mouse models by increased leakage of molecules into the brain parenchyma, loss of tight-junctions and reduced pericyte coverage [41–43].

To further elucidate the underlying mechanism regarding the observed age-effect on increased GBCA invasion into the brain parenchyma, we performed western blot analysis to quantify occludin protein expression, a marker for BBB-integrity [44, 45]. Although we saw a slight trend towards lower occludin expression in tg mice, we could only analyse the occludin expression at the final experimental time point (at an age of 12 months), which prevented us from testing the previously important effect of age on occludin expression. These findings indicate that while aging and AD pathology in tg mice may enhance BBB permeability or solute entry into parenchyma, the capacity for evasion of contrast agent (as a proxy for clearance) remained unaffected within the tested mice. Nevertheless, it should be noted that the abundance of tight-junction proteins does not always map linearly onto functional BBB permeability. Total occludin levels can fluctuate through post-translational redistribution, phosphorylation, and internalization without a corresponding change in paracellular permeability, as reviewed by Shigetomi and Ikenouchi [46]. Therefore, the observed trend in occludin expression, while suggestive, is not sufficient on its own to infer a definitive functional barrier change.

Within the experimental framework, MRI measurements were carried out under isoflurane anaesthesia which could impact cerebral hemodynamic and the glymphatic system. A comprehensive analysis of cerebral vasculature dilation and cerebral blood flow increase due to isoflurane anaesthesia [47] might hints towards an artificially high GBCA-influx in our model as a possible limitation. Previous data from MRI experiments using intracisternal tracer injections showed reduced CSF inflow into brain parenchyma and increased elimination via alternative routes especially around cranial nerves, the cribriform plate and cervical lymph nodes in anesthetized mice [48]. However, as tg mice and their wildtype littermates followed the same anaesthesia protocol, an effect on genotype differences should be negligible. Nevertheless, the prolonged duration of anaesthesia may have compressed the overall dynamic range of clearance and contributed to the incomplete washout of the contrast agent, limiting the absolute interpretation of the observed kinetics. Importantly, the animals remained physiologically stable during the measurements, as evidenced by a constant heart rate, indicating that the results were not confounded by instability. Other possible factors that could have contributed to an increased GBCA-influx could include altered aquaporin 4 (AQP4) polarization at astroglial endfeet [49] and could be a promising target for future studies.

Given that this model more closely reflects hereditary forms of AD based on Aβ overproduction, this is not entirely surprising but an important characteristic of the model that needs to be considered in future studies. However, the observation of manifest Aβ-pathology and neuroinflammation without overt brain-clearance impairment hints toward a crucial insight: impairment of brain clearance might not cause pathogenic Aβ deposition and subsequent neuroinflammation (starting at early disease stages) but rather is a consequence of them at later stages than the pathology observed in our mice. These findings may contribute to the ongoing discussion regarding the temporal relationship between amyloid pathology and clearance impairment. Future studies employing higher-resolution imaging and alternative tracers may help to further substantiate this intriguing line of thought with additional evidence.

As a limitation of our study, the absence of an effect may reflect a type II error due to limited power with small sample sizes. The number of mice used in the experiments was estimated as low as possible in accordance with the reduction aspect of the 3R principles of animal research that might have limited our statistical power. For the primary hypothesized reduced clearance efficacy in tg mice, by comparison of ventricular decay-constant c2, the calculated effect size was small (Cohen’s d = 0.254). Consequently, while major clearance deficits are unlikely, smaller biologically relevant effects may have remained undetected. Our results suggest that the genotype has only a minor impact on major integrative measurements of brain clearance within the experimental set up, potentially explaining why no significant difference was detected despite the advanced AD pathology.

## Conclusions

In summary, our findings suggest that in the examined tg mice, no evidence of impaired clearance was detected despite pre-existing amyloid pathology and neuroinflammation at six, nine, and twelve months of age. While this result contradicts our primary hypothesis of reduced brain clearance in aged tg mice, it provides a critical nuance to the understanding of AD pathogenesis using these APPswe/PS1dE9 AD murine model.

## Supplementary Information

Below is the link to the electronic supplementary material.

Supplementary Material 1: Supplementary Figure 1: Examples for histological quantifications. 6E10+ Plaque size (yellow polygon) used for quantification in histological samples.

Supplementary Material 2: Supplementary Figure 2: Representative transverse mouse brain sections used for histological analyses of fluorescent dye dispersion. Consecutive sections were collected at 210 µm intervals, as described in the Methods (2.3.2). The approximate anatomical localization of each section was determined using The Mouse Brain in Stereotactic Coordinates [50]. Due to tissue shrinkage and compression occurring during tissue processing and section preparation, the anatomical levels shown do not perfectly correspond and should be regarded as approximate.

Supplementary Material 3: Supplementary Figure 3: Example of the fitted model for curve analysis. Image of the measured time intensity curves (as % change of pixel value compared to baseline (black)) and the single-model-function (red) used to calculate the exponential decay-constants “c1” and “c2” using IgorPro V6.37

Supplementary Material 4: Supplementary Figure 4: Corresponding images of wildtype mice at the age of 6, 9 and 12 months in immunohistochemical specimen to visualize Amyloid β plaques (6E10+). Scale bar 50 µm

Supplementary Material 5: Supplementary Figure 5: Corresponding images of wildtype mice at the age of 6, 9 and 12 months in immunohistochemical specimen to visualize Iba1+ cells. Scale bar 50 µm

Supplementary Material 6: Supplementary Figure 6: Individual curves of % change of pixel value (as a measurement for signal intensity in the respective volumes of interest (VOIs). A) and D) both lateral ventricles, B) and E) 3rd Ventricle, C) and F) 4th Ventricle compared to baseline in wt (blue) and tg mice (red) at nine (left) and 12 months of age (right).

Supplementary Material 7: Supplementary Figure 7: Stable heartbeat rates in APPswe/PS1dE9 (tg, red) mice and their wildtype (wt, blue) littermates at 9 and 12 months of age during invasion phase of the GBCA during MRI measurements. Data presented as mean ± standard deviation of n = 9 wt and n = 9 tg mice at 9 months and n = 9 wt and n = 8 tg mice at 12 months.

Supplementary Material 8: Supplementary Figure 7: Stable heartbeat rates in APPswe/PS1dE9 (tg, red) mice and their wildtype (wt, blue) littermates at 9 and 12 months of age during invasion phase of the GBCA during MRI measurements. Data presented as mean ± standard deviation of n = 9 wt and n = 9 tg mice at 9 months and n = 9 wt and n = 8 tg mice at 12 months.

Supplementary Material 9

## Data Availability

The datasets used and/or analysed during the current study are available from the corresponding author on reasonable request.

## Abbreviations

AD: Alzheimer’s disease
APP: Amyloid precursor protein
Aβ: Amyloid β
BBB: Blood-brain barrier
BCSFB: Blood-cerebrospinal fluid barrier
CSF: Cerebrospinal fluid
FLAIR: Fluid attenuation inversion recovery
GBCA: Gadolinium-based contrast agents
ISF: Interstitial fluid
LALLF: Landesamt für Landwirtschaft, Lebensmittelsicherheit und Fischerei
MRI: Magnetic resonance imaging
PS: Presenilin
RARE: Rapid acquisition with relaxation enhancement
tg: Transgene APPswe/PS1dE9
wt: Wildtype
