# Supplementary material for: No evidence of major integrative brain clearance impairment in an Alzheimer´s disease model of transgenic APPswe/PS1dE9 mice
Source: Fluids Barriers CNS. 2026 Sep 5;23:107. doi: 10.1186/s12987-026-00872-9 (PMC13551768; doi:10.1186/s12987-026-00872-9)
Supplement: Supplementary file 1 [file 12987_2026_872_MOESM9_ESM.docx]

**Supplementary material**

*Supplementary Code 1:*

run("Set Scale...", "distance=726 known=1000 pixel=1 unit=µm");

run("8-bit");

run("HiLo");

run("Brightness/Contrast...");

setMinAndMax(55,255);

run("Apply LUT");

run("Close");

setOption("BlackBackground", false);

run("Make Binary");

run("Fill Holes");

run("Analyze Particles...", "size=500-Infinity pixel circularity=0.00-1.00 show=[Overlay Masks] display include summarize add");

**Supplementary Figure 1: Examples for histological quantifications.**

6E10^+^ Plaque size (yellow polygon) used for quantification in histological samples.

**Supplementary Figure 2:** **Representative transverse mouse brain sections used for histological analyses of fluorescent dye dispersion.**

Consecutive sections were collected at 210 µm intervals, as described in the Methods (2.3.2). The approximate anatomical localization of each section was determined using The Mouse Brain in Stereotactic Coordinates (50). Due to tissue shrinkage and compression occurring during tissue processing and section preparation, the anatomical levels shown do not perfectly correspond and should be regarded as approximate.

**Supplementary Figure 3: Example of the fitted model for curve analysis.**

Image of the measured time intensity curves (as % change of pixel value compared to baseline (black)) and the single-model-function (red) used to calculate the exponential decay-constants “c1” and “c2” using IgorPro V6.37

**Supplementary Figure 4: Corresponding images of wildtype mice at the age of 6, 9 and 12 months in immunohistochemical specimen to visualize Amyloid β plaques (6E10^+^)**

Scale bar 50 µm

**Supplementary Figure 5: Corresponding images of wildtype mice at the age of 6, 9 and 12 months in immunohistochemical specimen to visualize Iba1^+^ cells**

Scale bar 50 µm

**Supplementary Figure 6: Individual curves of % change of pixel value (as a measurement for signal intensity in the respective volumes of interest (VOIs)**

A) and D) both lateral ventricles, B) and E) 3rd Ventricle, C) and F) 4th Ventricle compared to baseline in wt (blue) and tg mice (red) at nine (left) and 12 months of age (right).

**Supplementary Figure 7: Stable heartbeat rates in APPswe/PS1dE9 (tg, red) mice and their wildtype (wt, blue) littermates at 9 and 12 months of age during invasion phase of the GBCA during MRI measurements.**

Data presented as mean ± standard deviation of n = 9 wt and n = 9 tg mice at 9 months and n = 9 wt and n = 8 tg mice at 12 months.

**Supplementary Figure 8: Western blots of occludin expression in the cortex and hippocampus from APPswe/PS1dE9 (tg) and wild-type (wt) mice.** The left panels show the occludin blots together with the corresponding gel images containing the molecular weight marker to indicate protein size. The right panels show the corresponding GAPDH housekeeping control blots with also gel images. Protein samples from individual animals (648 [tg], 653 [wt], 664 [tg], 657 [wt], 670 [tg], 658 [wt], 672 [tg], 666 [wt], 545 [tg], 550 [wt], 546 [tg], 554 [wt], 549 [tg], 556 [wt], 563 [tg], and 564 [wt]) were separated by SDS–PAGE and transferred to membranes for immunoblotting. Molecular weight standards are indicated in kDa. On the lowest and second lowest blots (Cortex/Hippocampus), samples from hippocampus were annotated with ‘Hip’ and samples from cortex with ‘Cx’ to better distinguish the samples.
